# Association of gut microbiota with glycaemic traits and incident type 2 diabetes, and modulation by habitual diet: a population-based longitudinal cohort study in Chinese adults

**DOI:** 10.1007/s00125-022-05687-5

**Published:** 2022-03-31

**Authors:** Huijun Wang, Wanglong Gou, Chang Su, Wenwen Du, Jiguo Zhang, Zelei Miao, Congmei Xiao, Zengliang Jiang, Zhihong Wang, Yuanqing Fu, Xiaofang Jia, Yifei Ouyang, Hongru Jiang, Feifei Huang, Li Li, Bing Zhang, Ju-Sheng Zheng

**Affiliations:** 1 Chinese Center for Disease Control and Prevention, National Institute for Nutrition and Health, Beijing, China; 2 Key Laboratory of Trace Element Nutrition, National Health Commission, Beijing, China; 3 Key Laboratory of Growth Regulation and Translational Research of Zhejiang Province, School of Life Sciences, Westlake University, Hangzhou, China; 4 Westlake Intelligent Biomarker Discovery Lab, Westlake Laboratory of Life Sciences and Biomedicine, Hangzhou, China; 5 Institute of Basic Medical Sciences, Westlake Institute for Advanced Study, Hangzhou, China

**Keywords:** Glycaemic traits, Gut microbiota, Longitudinal cohort, Type 2 diabetes

## Abstract

**Aims/hypothesis:**

The gut microbiome is mainly shaped by diet, and varies across geographical regions. Little is known about the longitudinal association of gut microbiota with glycaemic control. We aimed to identify gut microbiota prospectively associated with glycaemic traits and type 2 diabetes in a geographically diverse population, and examined the cross-sectional association of dietary or lifestyle factors with the identified gut microbiota.

**Methods:**

The China Health and Nutrition Survey is a population-based longitudinal cohort covering 15 provinces/megacities across China. Of the participants in that study, 2772 diabetes-free participants with a gut microbiota profile based on 16S rRNA analysis were included in the present study (age 50.8 ± 12.7 years, mean ± SD). Using a multivariable-adjusted linear mixed-effects model, we examined the prospective association of gut microbiota with glycaemic traits (fasting glucose, fasting insulin, HbA_1c_ and HOMA-IR). We constructed a healthy microbiome index (HMI), and used Poisson regression to examine the relationship between the HMI and incident type 2 diabetes. We evaluated the association of dietary or lifestyle factors with the glycaemic trait-related gut microbiota using a multivariable-adjusted linear regression model.

**Results:**

After follow-up for 3 years, 123 incident type 2 diabetes cases were identified. We identified 25 gut microbial genera positively or inversely associated with glycaemic traits. The newly created HMI (per SD unit) was inversely associated with incident type 2 diabetes (risk ratio 0.69, 95% CI 0.58, 0.84). Furthermore, we found that several microbial genera that were favourable for the glycaemic trait were consistently associated with healthy dietary habits (higher consumption of vegetable, fruit, fish and nuts).

**Conclusions/interpretation:**

Our results revealed multiple gut microbiota prospectively associated with glycaemic traits and type 2 diabetes in a geographically diverse population, and highlighted the potential of gut microbiota-based diagnosis or therapy for type 2 diabetes.

**Data availability:**

The code for data analysis associated with the current study is available at https://github.com/wenutrition/Microbiota-T2D-CHNS

**Graphical abstract:**

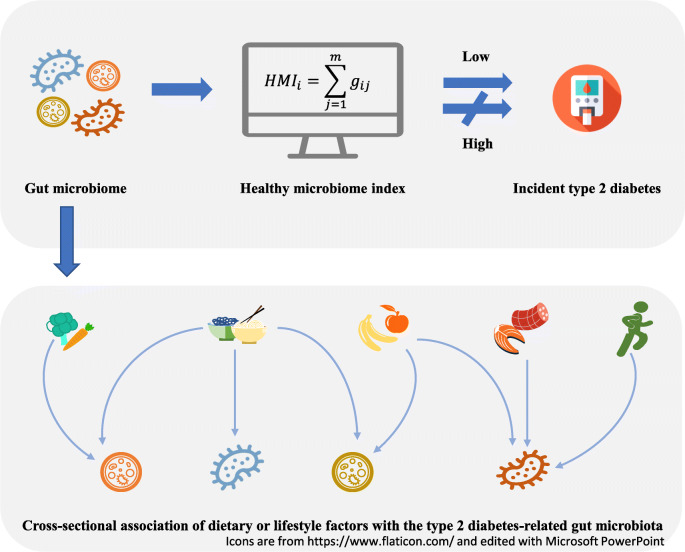

**Supplementary Information:**

The online version contains peer-reviewed but unedited supplementary material available at 10.1007/s00125-022-05687-5.

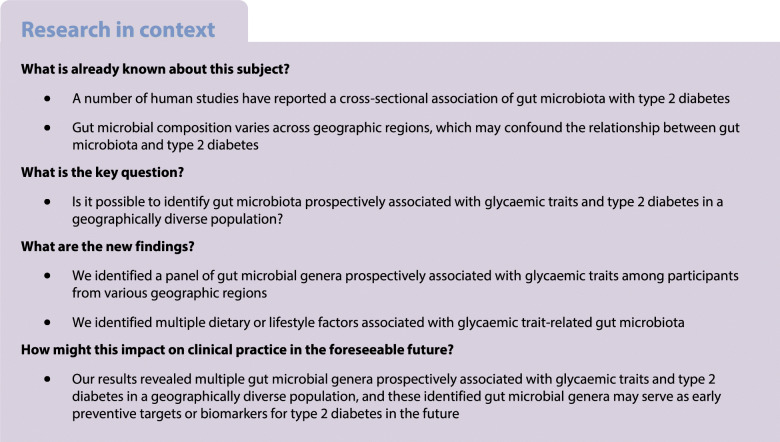



## Introduction

Type 2 diabetes results in a huge social and economic burden for society, and its prevalence has continued to increase globally in the past decade [1]. Gut microbiota interact with dietary constituents, producing choline, phenols, bile acids and short-chain fatty acids. These microbiota-derived metabolites may play vital roles in modulating the development of host metabolic diseases, including type 2 diabetes [2, 3]. Gut microbial markers are potential interventional targets for the prevention of type 2 diabetes [4]. Several human studies have reported a cross-sectional association of the microbiota with type 2 diabetes [3, 5, 6]. Recently, two European cohorts with relatively moderate sample sizes (*n* = 273 and 608, respectively) examined the prospective association of the gut microbiota with type 2 diabetes or glycaemic traits [7, 8]. However, the results from these previous studies were inconsistent, and evidence from large prospective cohorts is still lacking.

Gut microbial composition varies across geographic regions, which may confound the relationship between gut microbiota and type 2 diabetes [9]. Studies covering participants from various geographic regions may capture the enormous heterogeneities in gut microbial composition and other environmental factors, and are therefore highly necessary in gut microbial research. In addition, the complex interaction between diet and the gut microbiome may play an important role in modulating the host’s metabolic health [10]. For example, dietary fibre may be fermented by specific gut microbes, generating short-chain fatty acids that stimulate the secretion of glucagon-like peptide-1 and regulate glucose metabolism [11]. Gut microbiota-targeted dietary intervention is a promising and cost-efficient method to reduce disease risk [12, 13]. However, neither of the prospective studies [7, 8] evaluated the association of dietary or lifestyle factors with gut microbial genera that were specifically associated with type 2 diabetes or glycaemic traits.

Therefore, using a population-based longitudinal cohort, the China Health and Nutrition Survey (CHNS), covering 15 provinces/megacities across China, we aimed to identify gut microbiota prospectively associated with glycaemic traits and type 2 diabetes. As a secondary objective, we aimed to identify potential dietary or lifestyle factors associated with the glycaemic trait-related gut microbiota.

## Methods

### Study design

The present study was based on data from the CHNS, a unique population-based longitudinal study in China that covers key phenotypes, diet and health outcomes of participants from 15 provinces or megacities in China (six in the Northern region, nine in the Southern region) [14]. The detailed study design of CHNS has been described previously [14]. CHNS rounds were completed in 1989, 1991, 1993, 1997, 2000, 2004, 2006, 2009, 2011, 2015 and 2018. Stool samples and dietary information were collected in the 2015 survey, and participants with a gut microbiota profile based on 16S rRNA analysis from stool samples were included in the present study (*n* = 3248). Participants were excluded if they had used antibiotics within the month preceding stool collection (*n* = 71), had ever had an intestinal disease (*n* = 26, including ulcerative colitis, Crohn’s disease, localised enteritis or irritable bowel syndrome), or had prevalent type 2 diabetes in 2015 (*n* = 379). Therefore, a total of 2772 diabetes-free participants from the 2015 survey for whom a gut microbiota profile was available were included in the present study (age 50.8 ± 12.7] years, mean ± SD). After a median follow-up period of 3.04 years (IQR 2.9–3.1 years), 1829 participants remained at the time of the 2018 survey, 123 of whom had incident type 2 diabetes. These participants were included in our longitudinal analysis of gut microbiota with glycaemic traits and incident type 2 diabetes.

The CHNS protocol was approved by the Institutional Review Boards of the Chinese Center for Disease Control and Prevention (number 201524), the University of North Carolina at Chapel Hill, USA, and the US National Institute for Nutrition and Health (number 07-1963). Informed consent was obtained from all participants.

### Faecal sample collection and 16S rRNA profiling

Stool samples were collected by the participants themselves, who received instruction for the collection process during a home visit on one of the two weekdays when the 24 h dietary recall data were recorded, and immediately frozen at −20°C after collection. All stool samples were transported through a cold chain to the central laboratory within 24–48 h and stored at −20°C until processing. We obtained a mean of 76,881 paired-end raw reads for each sample. The methods for DNA extraction, amplification and sequencing have been described previously [15]. The 16S rRNA sequencing data were analysed using the Quantitative Insights Into Microbial Ecology 2 platform (QIIME 2) [16]. DADA2 software [17] was used to filter out sequencing reads with quality score *Q*<25 and to de-noise reads into amplicon sequence variants, resulting in feature tables and representative sequences. Taxonomy classification was performed based on the naive Bayes classifier using the classify-sklearn package against the Silva-132-99 reference sequences [18].

### Data collection

Demographic, lifestyle and dietary data were collected by questionnaires during the home visits on three consecutive days. Anthropometric factors were measured on-site by trained staff. Habitual dietary and total energy intakes were assessed by three consecutive 24 h dietary recalls, including two weekdays and one weekend day. The participants were asked to report the types and amounts of all food eaten during the previous 24 h [19]. The energy intake was calculated from the collected dietary data based on the Chinese Food Composition Table [20]. Physical activity was assessed as a total metabolic equivalent for task hours per week from 7-day recalls of occupational, transportation, domestic and leisure activities [21]. Urbanisation was quantified by a validated index covering 12 urbanicity-related components [22]. We assessed household income as the total income of all household members.

Following an overnight fast, a blood sample was collected by venepuncture. Blood glucose levels were measured using a glucose oxidase phenol 4-aminoantipyrine peroxidase kit (Randox, Crumlin, UK) and a Hitachi 7600 Analyzer (Hitachi, Tokyo, Japan). Serum insulin levels were measured using a radioimmunology assay kit (North Institute of Biological Technology, Beijing, China) and a XH-6020 gamma counter (North Institute of Biological Technology). HPLC (model HLC-723 G7; Tosoh Corporation, Tokyo, Japan) was used to measure HbA_1c_ [23]. The coefficients of variation for fasting glucose, insulin and HbA_1c_ at follow-up were 19%, 13% and 16%, respectively. HOMA-IR (calculated as fasting glucose × fasting insulin/22.5) was used to represent insulin resistance.

### Ascertainment of type 2 diabetes

Incident type 2 diabetes cases were ascertained based on fasting blood glucose ≥7.0 mmol/l or HbA_1c_ ≥47.5 mmol/mol (6.5%), or being currently under medical treatment for diabetes during the follow-up visits, according to the American Diabetes Association criteria for the diagnosis of diabetes [24].

### Bioinformatics and statistical analysis

Statistical analyses were performed using Stata 15 (StataCorp, College Station, TX, USA). The classifier was based on codes adapted from the scikit-learn package [25]. Missing values of the continuous covariates were imputed from the mean value in the corresponding regions (i.e. North or South China), and categorical covariates were imputed from the highest frequency value. Only microbial genera present in at least 10% of the participants were included in our analyses.

#### Comparison of the gut microbial composition between participants from North and South China

At the genus level, we used the *vegdist* function from the R package vegan [26] to calculate the gut microbial Bray–Curtis dissimilarity matrix. The *p* value was determined by 1000 permutations, and a *p* value <0.05 was considered statistically significant.

A machine learning model (gradient boosting decision trees from the Light Gradient Boosting Machine [LightGBM] package [27]) was used for classification of participants from North or South China. The genus-level taxonomic abundance was used as the predictive feature. We used the ‘leave one out’ strategy to evaluate the classifier’s performance, meaning that each training set was created by taking all provinces or megacities except for the test set. The above process was repeated ten times, resulting in a probability for each participant belong to the Southern region.

We used the SHAP (Shapley Additive exPlanations) algorithm [28] to estimate the contribution of each gut microbial genus to the overall classifier prediction. Combination of the LightGBM and SHAP method has shown unique strength in prediction and feature selection [6, 29]. Microbial genera with a mean absolute SHAP value greater than 0 contributed to the classification of geographic regions, and were treated as a region-discriminating gut microbe.

### Region-discriminating gut microbiota predicted dietary habits

For each of the dietary factors, we used the LightGBM method to predict the dietary intake based on the region-discriminating microbial genera. The tested dietary factors including rice, wheat, fruit, vegetable, nuts, pork, poultry, milk, egg, fish, animal oil and vegetable oil. We constructed an index by generating the wheat/rice ratio to reflect the staple food preference. A tenfold cross-validation predictive implementation was used to generate genera-predicted intake values for each participant. The performance of the model was quantified using Pearson correlation for regression and the AUC of the receiver operating characteristic for classification. The R package pROC [30] was used for receiver operating characteristic curve analyses. As a sensitivity analysis, we also imputed the missing dietary factors by multiple imputation using chained equations. The multiple imputation model included the outcome (dietary factors), age, sex, education, marital status, education and geographic region (North or South China). Five imputed datasets were generated, and the prediction analyses were based on the mean values of the imputed datasets.

#### Longitudinal relationship between gut microbiota and glycaemic traits

At the genus level, we used a linear mixed-effects model to examine the longitudinal association of gut microbiota with glycaemic traits (fasting glucose, fasting insulin, HbA_1c_ and HOMA-IR), adjusted for the corresponding baseline glycaemic trait, demographic, anthropometric and lifestyle factors. Sensitivity analysis was performed by adding the dietary factors into the covariate list. The demographic, anthropometric and lifestyle factors included age, sex, household income, marital status, self-reported educational level, place of residence (rural or urban), urbanisation index, BMI, total energy intake, alcohol consumption, smoking and physical activity. To further identify microbial genera associated with glycaemic traits that are potentially mediated by BMI, we re-examined the association of the gut microbiota with glycaemic traits without adjusting for the BMI. Here, associations were expressed as the difference in glycaemic traits (in SD units) per SD difference in each gut microbial genus. The linear mixed-effects model contains a random intercept and random coefficient on the provinces or megacities to adjust for the heterogeneity of the gut microbiota composition among the provinces or megacities. We independently examined the gut microbiota/glycaemic trait association in the Northern and Southern populations, and combined the effect estimates from the two regions using random-effects meta-analysis. A *p* value <0.05 was considered statistically significant. The Benjamini–Hochberg method was used to control the false discovery rate (FDR).

#### Healthy microbiome index and incident type 2 diabetes

We used an additive model to construct a healthy microbiome index (HMI) with the glycaemic trait-related genera as

$$ {\mathrm{HMI}}_i=\sum \limits_{j=1}^m{g}_{ij} $$

where HMI_*i*_ is a healthy microbiome index for individual *i*, *m* is the number of glycaemic trait-related genera, and *g*_*ij*_ is the score for gut microbial genus *j* for the individual *i*. If the individual *i* carries genus *j* that is in favour of a glycaemic trait, or does not carry genus *j* that is harmful to the glycaemic trait, *g*_*ij*_ equals 1, otherwise *g*_*ij*_ equals 0.

We then examined the prospective association of the baseline HMI (per SD unit) with incident type 2 diabetes using a Poisson regression model, adjusted for the aforementioned demographic, anthropometric and lifestyle factors. We also performed subgroup analysis stratified by the geographic region, age group, sex, BMI level and urbanisation level (city or rural), to test the robustness of the model.

#### Relationship between dietary or lifestyle factors and glycaemic trait-related gut microbiota

Linear regression was used to estimate the difference in the above glycaemic trait-related gut microbiota or HMI (in SD units) per SD change for continuous dietary or lifestyle factors (per-category change for categorical dietary or lifestyle factors), with adjustment for potential confounders and mutually adjusted for the other dietary or lifestyle factors. The tested dietary or lifestyle factors included wheat, rice, wheat/rice ratio, fruit, vegetable, nuts, pork, poultry, milk, egg, fish, alcohol consumption, smoking and physical activity. The adjusted covariates included age, sex, BMI, total energy intake, household income, marital status, self-reported educational level, place of residence (rural or urban), urbanisation index, and animal or vegetable oil intake. In addition to the above food groups, we also used linear regression to evaluate the association of dietary fibre with glycaemic trait-related gut microbial genera, with adjustment for the above covariates. The Benjamini–Hochberg method was used to control the FDR. An FDR value <0.05 was considered statistically significant. We further used linear regression to examine the association between the included food groups and glycaemic traits with and without adjustment for the gut microbial genera (i.e. HMI).

## Results

### Participant characteristics

**Fig. 1:**
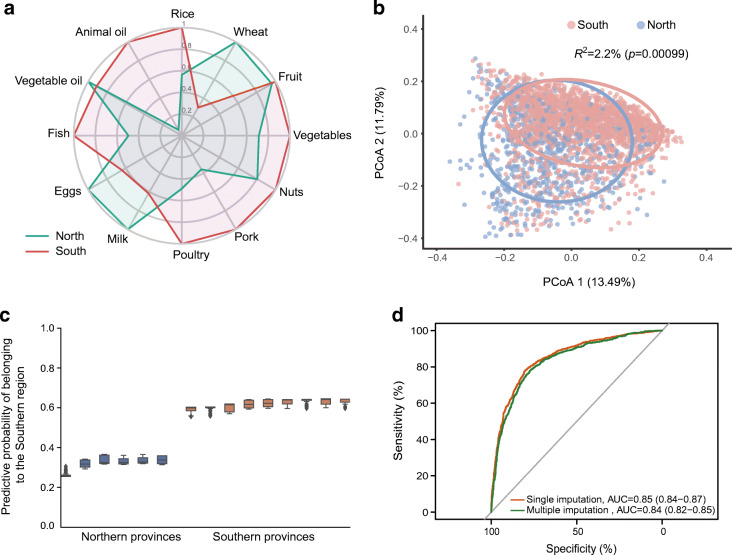
Region-discriminating gut microbiota and dietary habits. (**a**) Comparison of dietary habits among participants from Northern and Southern China (*n* = 2772). For each dietary factor, data are presented as scaled mean values (i.e. mean values divided by the corresponding maximum mean value of two regions). (**b**) Dissimilarities in gut microbial composition between participants from Northern and Southern China represented by a Bray–Curtis dissimilarity matrix and principal coordinate analysis. The *p* value was determined by 1000 permutations. The level of confidence for the ellipses was 85%. The values on the axes represent the variance of the gut microbial composition at the genus level explained by principal components PCoA1 and PCoA2. (**c**) The microbial genera-based classifier achieved a high performance in regional prediction. The genus-level taxonomic abundance was used as the predictive features for the LightGBM model to predict the probability for each participant of belonging to the Southern region. (**d**) Receiver operator characteristic curves classifying participants’ staple food preference. We used the region-discriminating genera as input for the LightGBM model to predict the staple food preference. Staple food preference was calculated as the ratio of wheat intake to rice intake. A ratio ≥1 was considered as a wheat preference, otherwise a rice preference was inferred. Here, missing values were imputed using strategies (single mean imputation and multiple imputation). AUC indicates a tenfold cross-validated AUC. The range shown by the AUC is the 95% CI of the receiver operator characteristic curves

The overview of the study workflow is shown in electronic supplementary material (ESM) Fig. 1. The proportions of prevalent and incident type 2 diabetes in Northern China were 12.8% and 6.73%, respectively, and 11.6% and 6.72%, respectively, in Southern China. Baseline characteristics of the CHNS study participants are shown in Table 1. The proportions of participants for whom data were missing were low, as shown in ESM Table 1. After excluding rare microbial genera that were present in less than 10% of all the participants, 191 gut microbial genera were included in our study.

**Table 1:** Characteristics of the participants included in this study

	Overall	Northern China	Southern China
Number of participants	2772	992	1780
Duration of follow-up, years	3.0 ± 0.09	3.0 ± 0.1	3.0 ± 0.07
Age, years	50.8 ± 12.7	50.9 ± 13.2	50.7 ± 12.5
Women, *n* (%)	1328 (47.9)	461 (46.5)	867 (48.7)
BMI, kg/m^2^	24.1 ± 3.3	24.8 ± 3.4	23.7 ± 3.2
Education, *n* (%)			
Middle school or lower	1801 (65.0)	616 (62.1)	1185 (66.6)
High school or professional college	608 (21.9)	215 (21.7)	393 (22.1)
University	363 (13.1)	161 (16.2)	202 (11.3)
Married, *n* (%)	2407 (86.8)	894 (90.1)	1513 (85.0)
Income (10,000 yuan/year per household)	7.3 ± 10.5	6.4 ± 8.6	7.8 ± 11.4
Urban, *n* (%)	954 (34.4)	315 (31.8)	639 (35.9)
Urbanisation index	72.5 ± 17.5	69.1 ± 18.1	74.5 ± 16.8
Physical activity, MET	147.0 ± 150.8	138.8 ± 141.7	151.6 ± 155.5
Mean daily energy intake, kJ/day	8316.9 ± 2837.5	8372.6 ± 2869.8	8285.9 ± 2819.6
Current smoking, *n* (%)	748 (27.0)	253 (25.5)	495 (27.8)
Current alcohol consumption, *n* (%)	819 (29.5)	284 (28.6)	535 (30.1)
Rice intake, kg/day	0.2 ± 0.2	0.2 ± 0.2	0.3 ± 0.1
Wheat intake, kg/day	0.1 ± 0.2	0.2 ± 0.2	0.07 ± 0.07
Fruit intake, kg/day	0.04 ± 0.07	0.04 ± 0.07	0.04 ± 0.06
Vegetable intake, kg/day	0.3 ± 0.1	0.2 ± 0.1	0.3 ± 0.1
Nut intake, kg/day	0.003 ± 0.009	0.003 ± 0.008	0.004 ± 0.01
Pork intake, kg/day	0.08 ± 0.07	0.04 ± 0.04	0.09 ± 0.07
Poultry intake, kg/day	0.02 ± 0.04	0.01 ± 0.03	0.02 ± 0.04
Milk intake, kg/day	0.01 ± 0.05	0.02 ± 0.07	0.01 ± 0.04
Egg intake, kg/day	0.03 ± 0.03	0.03 ± 0.04	0.02 ± 0.02
Fish intake, kg/day	0.03 ± 0.04	0.02 ± 0.03	0.03 ± 0.05
Vegetable oil intake, kg/day	0.02 ± 0.03	0.02 ± 0.02	0.02 ± 0.03
Animal oil intake, kg/day	0.004 ± 0.01	0.0004 ± 0.003	0.007 ± 0.01
Fasting glucose, mmol/l	5.2 ± 0.6	5.3 ± 0.6	5.1 ± 0.6
HbA_1c,_ mmol/mol	36.6 ± 4.3	36.7 ± 4.1	36.6 ± 4.4
HbA_1c_, %	5.5 ± 0.4	5.5 ± 0.4	5.5 ± 0.4
Fasting insulin, pmol/l	50.7 ± 44.1	47.4 ± 36.2	52.4 ± 47.5
HOMA-IR	1.7 ± 1.5	1.6 ± 1.3	1.7 ± 1.6

Data are presented as number of participants (%) or mean ± SD

MET, metabolic equivalent of task hours per week

### Dietary habits and gut microbial composition among participants from North and South China

The dietary habits and gut microbial composition showed strong geographic differences between Northern and Southern China (Fig. 1a,b). Participants from Northern China had a high consumption of wheat-based foods, milk and egg, while those from Southern China consumed more rice-based foods, animal oil, fish, vegetables, nuts, pork and poultry (Fig. 1a). The absolute values for the significant Pearson’s correlation coefficients within the dietary factors were low to moderate (ESM Table 2, 0.041≤ |r| ≤0.366).

The genera-based classifier showed a high performance for regional prediction (Fig. 1c). We identified 46 region-discriminating gut microbial genera (ESM Table 3) that contributed to the classification of Northern or Southern China based on the SHAP method. Several food groups (wheat, rice, pork) showed moderate to high correlation (Pearson’s correlation coefficients >0.15) in the tenfold cross-validation between predicted dietary values (based on region-discriminating gut microbiota) and measured dietary values (ESM Table 4). Further analysis showed that the region-discriminating gut microbial genera could predict a participant’s staple food preference (Fig. 1d).

### Prospective association of gut microbiota with glycaemic traits and type 2 diabetes

**Fig. 2:**
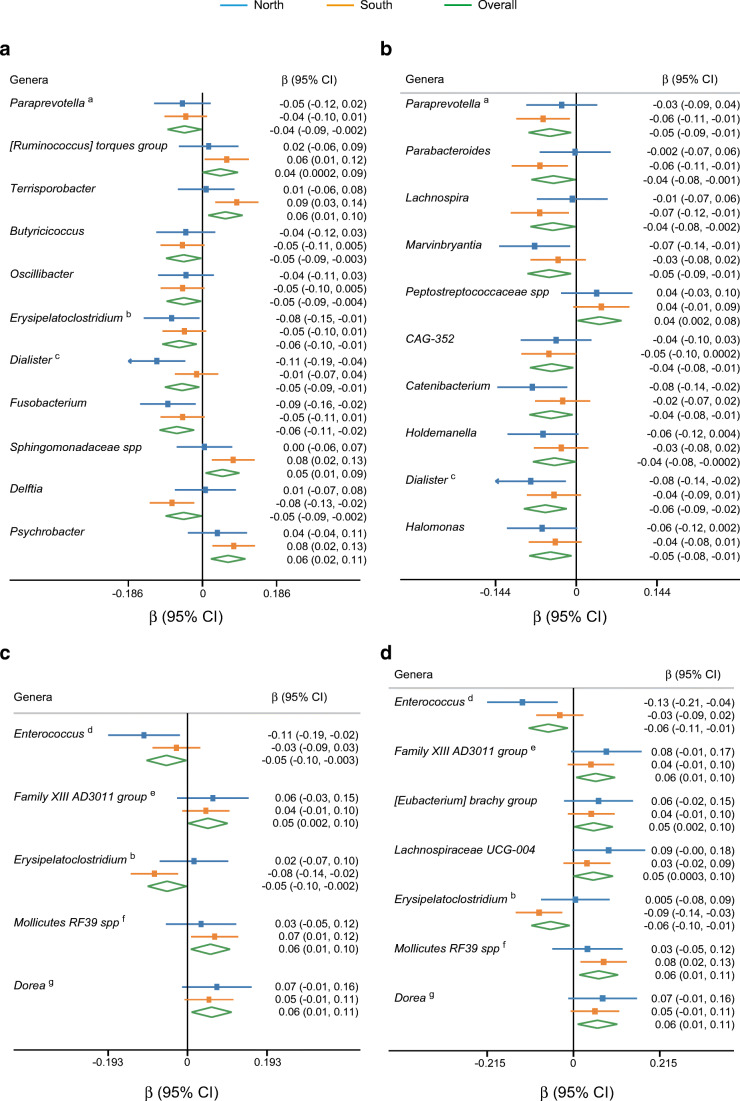
Prospective association between the gut microbiota and glycaemic traits. Prospective association of baseline gut microbiota with (**a**) fasting glucose, (**b**) HbA_1c_, (**c**) fasting insulin and (**d**) HOMA-IR. A total of 1829 participants were included in this analysis. A linear mixed-effects model was used to examine the prospective association of gut microbiota with the glycaemic traits fasting glucose, HbA_1c_, fasting insulin and HOMA-IR, adjusting for the baseline glycaemic traits, demographic, anthropometric and lifestyle confounders. We independently examined the gut microbiota/glycaemic trait association in the Northern and Southern populations, and combined the effect estimates from the two regions using random-effects meta-analysis. Associations are expressed as the difference in glycaemic traits (in SD units) per SD difference for each genus. Superscript letters (a to g) indicate that the marked gut microbial genera were associated with at least two glycaemic traits. A *p* value <0.05 was considered as statistically significant. No individual gut microbial genera were found to be associated with glycaemic traits after adjusting for multiple testing

**Fig. 3:**
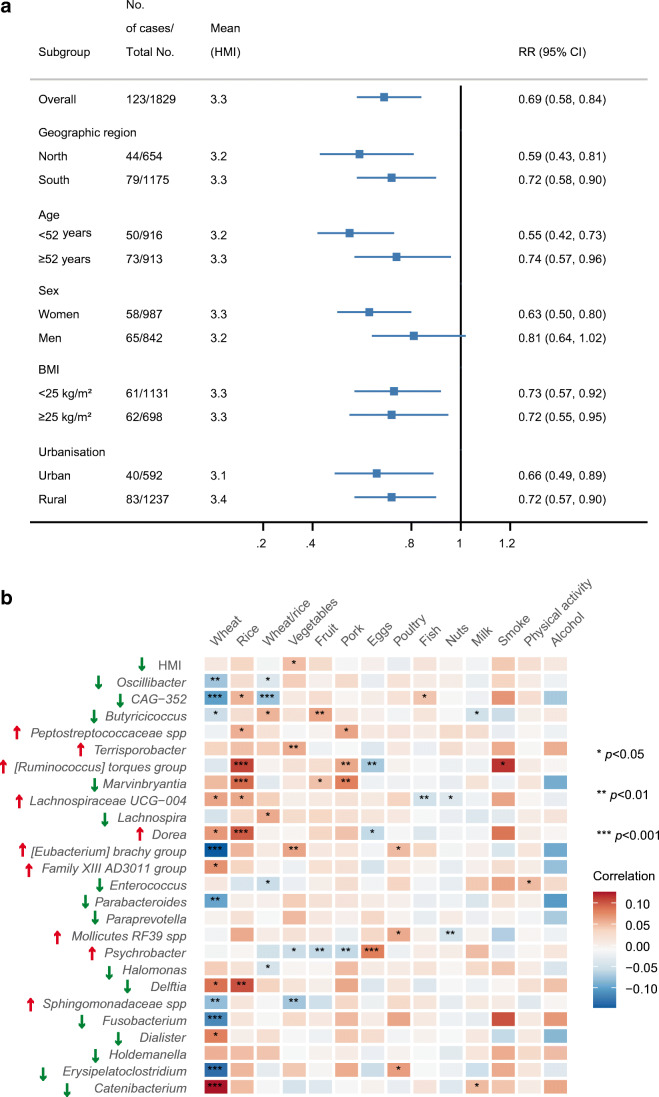
Association of HMI with incident type 2 diabetes and modulation by dietary and lifestyle factors. (**a**) HMI and type 2 diabetes incidence (*n* = 1829). Poisson regression was used to examine the association of baseline HMI (per SD unit) with incident type 2 diabetes, adjusted for demographic, anthropometric, dietary and lifestyle factors. Subgroup analyses stratified by geographic region, age group, sex, BMI level and urbanisation level (city or rural) were performed to test the robustness of the model. (**b**) Association of dietary and lifestyle factors with gut microbiota (*n* = 2772). Linear regression was used to estimate the difference in glycaemic trait-related gut microbiota or HMI (in SD units) per SD change for continuous dietary or lifestyle factors (per-category change for categorical dietary or lifestyle factors), with adjustment for the confounders and mutually adjusted for the other tested dietary or lifestyle factors. Red arrows indicate gut microbiota that were positively associated with glycaemic traits; green arrows indicate gut microbiota that were inversely associated with glycaemic traits. The Benjamini–Hochberg method was used to control the FDR. An FDR value <0.05 was considered statistically significant

Overall, a total of 25 gut microbial genera were positively or inversely associated with at least one glycaemic trait (Fig. 2), including seven region-discriminating genera (*Erysipelatoclostridium*, *Dialister*, *Fusobacterium*, [*Ruminococcus*] *torques group*, *Lachnospira*, *Marvinbryantia* and *Catenibacterium*). Similar results were obtained after further adjustment for dietary factors in the sensitivity analysis (ESM Fig. 2). However, no individual gut microbial genera were found to be associated with glycaemic traits after adjusting for multiple testing. Most identified genera had a high prevalence in our cohort (ESM Table 5, mean 55%). Seven of the 25 genera were consistently associated with at least two glycaemic traits, including *Erysipelatoclostridium*, *Dialister*, *Mollicutes RF39* spp., *Paraprevotella*, *Enterococcus*, *Family XIII AD3011 group* and *Dorea*. In addition to the above microbial genera, we identified additional genera that were inversely associated with glycaemic traits, including *Atopobium*, *Anaerofustis* and *Defluviitaleaceae UCG-011* in our model without adjustment for the BMI (ESM Table 6). We found that the HMI (per SD unit) showed an inverse association with incident type 2 diabetes (risk ratio 0.69, 95% CI 0.58,0.84) (Fig. 3a). Subgroup analysis showed similar results (Fig. 3a).

### Association of dietary and lifestyle factors with glycaemic trait-related microbial genera

A total of 53 pairs of dietary (or lifestyle) factor/gut microbiota associations were identified after multiple testing correction (Fig. 3b). Most glycaemic trait-related gut microbial genera were associated with at least one dietary or lifestyle factor (23/25). Overall, vegetable intake was positively associated with HMI. However, when considering the specific glycaemic trait-related bacteria, vegetable intake was significantly associated with a higher abundance of two genera (*Terrisporobacter* and [*Eubacterium*] *brachy group*) (Fig. 3b), which were unfavourable for the glycaemic trait. Wheat intake was significantly associated with 14 of the 25 glycaemic trait-related microbial genera. High intake of wheat was not consistently associated with genera, which were favourable or unfavourable for the glycaemic trait (Fig. 3b). High intakes of fruit, fish and nuts were consistently associated with lower abundance of the glycaemic trait-positive associated genera, and higher abundance of the glycaemic trait-negative associated genera. None of the gut microbial genera were associated with fibre intake after adjustment for multiple testing. We obtained similar results for the associations between dietary factors and glycaemic traits with and without adjustment for glycaemic trait-related microbial genera (ESM Fig. 3).

## Discussion

In this longitudinal cohort study, we demonstrated a large variability in the composition of gut microbiota between participants from Northern and Southern regions of China, and found that the geographic variation in gut microbiota was highly associated with habitual diet, especially the staple food preference of the participants. We identified key gut microbial genera, and created a new microbial index prospectively associated with type 2 diabetes among participants from the two geographic regions. We found multiple dietary or lifestyle factors associated with the identified gut microbial genera.

China is divided into Northern and Southern regions by the Qinling Mountains/Huai River line. Gut microbiota-based region classifiers worked well in our present study, suggesting that gut microbiota in the Northern and Southern China populations were notably different. Wheat (*Triticum* spp.) and rice (*Oryza sativa* var. *sinica*) are generally considered as the main staple foods in China, accounting for a high proportion of the daily diet in Northern and Southern China, respectively. The majority of the dietary wheat and rice were refined grains. Wheat contains about 1.7% (dry matter) non-digestible carbohydrates, mainly as xylose and arabinose, while rice contains 0.2% non-digestible carbohydrates [31]. A previous intervention study suggested that the staple foods, especially wheat, may rapidly alter gut microbial community structure and metabolic pathways [31]. The long-term differences in staple food preferences may shape the distinct gut microbial structures of the participants from Northern and Southern China.

In our present study, we identified a panel of microbial genera that were prospectively associated with the glycaemic traits. No individual gut microbial genera were found to be associated with glycaemic traits after adjusting for multiple testing. Most of the identified genera–glycaemic trait associations were first reported in a prospective study. The region-discriminating genus *Erysipelatoclostridium* was inversely associated with fasting glucose, insulin and insulin resistance in our study, consistent with a previous study showing that *Erysipelatoclostridium* was positively correlated with the glucose-lowering effects of metformin in humans [32]. Our results for *Dorea* are consistent with results from several cross-sectional studies that reported a positive relationship between *Dorea* and type 2 diabetes [33, 34]. It has been suggested that *Parabacteroides* is a beneficial commensal microbe producing short-chain fatty acids, which are beneficial for glucose metabolism [35]. In line with that study, our results showed that *Parabacteroides* was inversely associated with HbA_1c_. *Parabacteroides* has been reported to be positively associated with type 2 diabetes in several cross-sectional studies [33, 36, 37]. However, previous human and animal studies have demonstrated that hypoglycaemic agents increase the abundance of *Parabacteroides* [37–40]. These results may support the hypothesis that enrichment of *Parabacteroides* in type 2 diabetes patients may be a result of the drug treatment.

We also confirmed several microbial genera–glycaemic trait associations that have been reported in a Finnish prospective study [8]. Specifically, in the Finnish study, *Paraprevotella*, [*Ruminococcus*] *torques group* and *Family XIII AD3011 group* were considered as the most predictive microbial biomarkers (three of the top five ranked) for type 2 diabetes-associated variables. A high abundance of *Paraprevotella* was inversely associated with HbA_1c_ levels. In agreement with these results, *Paraprevotella* was negatively associated with fasting glucose and HbA_1c_ in our study. In the Finnish study, the [*Ruminococcus*] *torques group* contributed to the prediction of fasting insulin. Similarly, one of the region-discriminating genera, [*Ruminococcus*] *torques group*, was positively associated with fasting glucose in our study. Overall, despite the different study designs, population ethnicities and analysis strategies between the present study and the Finnish study, several microbial signatures were consistently associated with the risk of type 2 diabetes in the two studies. Additionally, we also identified some microbial genera (*Atopobium*, *Anaerofustis* and *Defluviitaleaceae UCG-011*) that were inversely associated with glycaemic traits potentially through BMI, although the detailed mechanism has yet to be determined. In support of this finding, a previous study found that *Atopobium* was inversely associated with BMI, and the abundance of *Atopobium* was higher in individuals with type 2 diabetes compared with healthy individuals [41].

Geographic variations in the gut microbial composition may limit application of a universal gut microbiota reference for diseases such as type 2 diabetes [9]. However, we demonstrated that the HMI was consistently associated with type 2 diabetes risk among participants from different geographic regions, age groups, sex, BMI levels and urbanisation levels. The strength of the current HMI was that it was developed and validated based on data from large national representative samples, and thus has high generalisability.

Understanding the role of habitual diet in gut microbiota is important for type 2 diabetes management and prevention [42]. Previous intervention studies have found that staple foods, especially wheat, effectively improved gut function and rapidly altered gut microbial community structure [31, 43]. In our study, wheat was associated with most genera that were favourable or unfavourable for glycaemic traits, highlighting the important role of wheat in gut microbial composition and glucose metabolism. An intervention study in humans found that grains such as whole-grain barley and brown rice reduced plasma interleukin-6 and glucose levels, and increased the abundance of *Dialister* [43]. In our study, *Dialister* showed a consistent inverse association with fasting glucose and HbA_1c_, and was positively associated with wheat intake. Overall, vegetable intake was positively associated with the HMI, suggesting that higher vegetable intake may help improve the gut microbiota profile. We also found several microbial genera that were favourable for glycaemic traits were consistently associated with healthy dietary habits (higher consumption of vegetables, fruit, fish and nuts). The associations of gut microbial genera with glycaemic traits remained largely unchanged after adjustment for dietary confounders. There are several possible reasons for this. On one hand, participants within each region may share similar dietary habits, and therefore the influence of dietary adjustment on the results may be attenuated as we evaluated the microbiota/glycaemic trait association in the Northern and Southern regions separately. On the other hand, many other lifestyle factors or even early-life factors such as delivery mode and maternal microbiota may also affect the abundance of gut microbiota [44].

This study has several strengths. First, as far as we are aware, it is the largest prospective study to date to investigate the association of gut microbiota with glycaemic traits and incident type 2 diabetes across geographic regions. In addition, we demonstrated that the inverse association of the HMI with type 2 diabetes was independent of the geographic region. This highlights the potential for gut microbiota-based diagnosis or therapy for type 2 diabetes across regions in China. Finally, we identified multiple dietary or lifestyle factors associated with glycaemic trait-related gut microbiota. A major limitation of the present study is that all participants included in the present study are Chinese, and caution should therefore be exercised in extrapolating our findings to other ethnic groups. Another limitation is that the gut microbes were measured only once and may not represent long-term status. Changes in gut microbes over time are likely. Finally, our analyses are based on genera rather than bacterial species. It is possible that different species within a genus may have different effects on glucose metabolism and different associations with dietary exposures.

In summary, we characterised the variations of gut microbiota among participants from Northern and Southern China. We identified a panel of gut microbiota that are prospectively associated with glycaemic traits and type 2 diabetes, and found several dietary and lifestyle factors to be associated with the identified specific gut microbial genera. The identified gut microbiota may serve as potential early preventive targets or biomarkers for type 2 diabetes.

## Supplementary Information

ESM 1 (PDF 280 kb)

## Data Availability

The data described in the article will be made available upon request pending application and approval. The code for data analysis associated with the current study is available at https://github.com/wenutrition/Microbiota-T2D-CHNS.

## Abbreviations

CHNS: China Health and Nutrition Survey
FDR: False discovery rate
HMI: Healthy microbiome index
LightGBM: Light Gradient Boosting Machine
SHAP: Shapley Additive exPlanations
