# Correction to: Association of gut microbiota with glycaemic traits and incident type 2 diabetes, and modulation by habitual diet: a population-based longitudinal cohort study in Chinese adults

**DOI:** 10.1007/s00125-022-05737-y

**Published:** 2022-06-20

**Authors:** Huijun Wang, Wanglong Gou, Chang Su, Wenwen Du, Jiguo Zhang, Zelei Miao, Congmei Xiao, Zengliang Jiang, Zhihong Wang, Yuanqing Fu, Xiaofang Jia, Yifei Ouyang, Hongru Jiang, Feifei Huang, Li Li, Bing Zhang, Ju-Sheng Zheng

**Affiliations:** 1 Chinese Center for Disease Control and Prevention, National Institute for Nutrition and Health, Beijing, China; 2 Key Laboratory of Trace Element Nutrition, National Health Commission, Beijing, China; 3 Key Laboratory of Growth Regulation and Translational Research of Zhejiang Province, School of Life Sciences, Westlake University, Hangzhou, China; 4 Westlake Intelligent Biomarker Discovery Lab, Westlake Laboratory of Life Sciences and Biomedicine, Hangzhou, China; 5 Institute of Basic Medical Sciences, Westlake Institute for Advanced Study, Hangzhou, China

**Correction to: Diabetologia**

10.1007/s00125-022-05687-5

Acknowledgment that this work was supported by the National Institutes of Health and National Institute of Diabetes and Digestive and Kidney Diseases (R01-DK104371) was omitted from the funding statement. The original article has been corrected.

