# Supplementary material for: Association of gut microbiota with glycaemic traits and incident type 2 diabetes, and modulation by habitual diet: a population-based longitudinal cohort study in Chinese adults
Source: Diabetologia. 2022 Mar 31;65(7):1145–56. doi: 10.1007/s00125-022-05687-5 (PMC9174105; doi:10.1007/s00125-022-05687-5)

Electronic Supplementary Material to:

"Association of gut microbiota with glycemic traits and incident type 2 diabetes, and modulation by habitual diet: a population-based longitudinal cohort study in Chinese adults"

ESM Tables

ESM Table 1. The missing rates of the covariates.

ESM Table 2. Pairwise correlation between dietary factors.

ESM Table 3. List of region-specific gut microbiota across the north and south China.

ESM Table 4. Region-specific gut microbiota predicted dietary habits.

ESM Table 5. Prevalence of the glycemic-related gut microbiota.

ESM Table 6. Prospective association of gut microbiota with glycemic traits

ESM Figures

ESM Fig. 1. Overview of the participants included in this analysis.

ESM Fig. 2. Prospective association of gut microbiota with the fasting glucose (A), HbA1c (B), fasting insulin (C) and HOMA IR (D).

ESM Fig. 3. Association of dietary factors with glycemic traits.

ESM Table 1. The missing rates of the covariates.

Variable	Missing rate
Income	2.27%
Smoke	0.04%
Alcohol	0.04%
Physical activity	0.07%
BMI	0.29%
Urbanisation index	1.23%
Rice intake	16.34%
Wheat intake	16.34%
Vegetable intake	16.34%
Fruit intake	16.34%
Nuts intake	16.34%
Pork intake	16.34%
Poultry intake	16.34%
Milk intake	16.34%
Egg intake	16.34%
Fish intake	16.34%
Vegetable oil intake	16.34%
Animal oil intake	16.34%
Energy intake	16.34%

ESM Table 2. Pairwise correlation between dietary factors*.

Food groups	Wheat	Rice	Fruit	Nuts	Pork	Poultry	Milk	Egg	Fish
Wheat	1.000								
Rice	-0.366***	1.000							
Fruit	-0.070***	-0.062***	1.000						
Nuts	-0.041*	-0.018	0.114***	1.000					
Pork	-0.289***	0.306***	0.046**	0.025	1.000				
Poultry	-0.144***	0.128***	0.039*	0.005	0.109***	1.000			
Milk	-0.065***	-0.117***	0.173***	0.053**	-0.067***	-0.018	1.000		
Egg	0.162***	-0.131***	0.103***	0.074***	-0.050**	-0.050**	0.060***	1.000	
Fish	-0.129***	0.011	0.161***	0.041**	0.058***	0.038*	0.082***	0.064***	1.000

*The correlation coefficients presented in the table were calculated by Pearson's method. *** $p<0.01$, ** $p<0.05$, * $p<0.1$

ESM Table 3. List of region-specific gut microbiota across the north and south China.

Taxa annotation
Bacteria;Firmicutes;Clostridia;Clostridiales;Ruminococcaceae;Ruminococcaceae UCG-013
Bacteria;Firmicutes;Clostridia;Clostridiales;Ruminococcaceae;[Eubacterium] coprostanoligenes group
Bacteria;Firmicutes;Erysipelotrichia;Erysipelotrichales;Erysipelotrichaceae;Catenibacterium
Bacteria;Firmicutes;Erysipelotrichia;Erysipelotrichales;Erysipelotrichaceae;Erysipelatoclostridium
Bacteria;Firmicutes;Negativicutes;Selenomonadales;Veillonellaceae;Dialister
Bacteria;Fusobacteria;Fusobacteriia;Fusobacteriales;Fusobacteriaceae;Fusobacterium
Bacteria;Actinobacteria;Actinobacteria;Actinomycetales;Actinomycetaceae;Actinomyces
Bacteria;Actinobacteria;Actinobacteria;Bifidobacteriales;Bifidobacteriaceae;Bifidobacterium
Bacteria;Proteobacteria;Alphaproteobacteria;Sphingomonadales;Sphingomonadaceae;Sphingomonas
Bacteria;Actinobacteria;Actinobacteria;Corynebacteriales;Nocardiaceae;Rhodococcus
Bacteria;Proteobacteria;Gammaproteobacteria;Betaproteobacteriales;Burkholderiaceae;Parasutterella
Bacteria;Verrucomicrobia;Verrucomicrobiae;Verrucomicrobiales;Akkermansiaceae;Akkermansia
Bacteria;Actinobacteria;Coriobacteriia;Coriobacteriales;Coriobacteriales Incertae Sedis;Raoultibacter
Bacteria;Actinobacteria;Coriobacteriia;Coriobacteriales;Eggerthellaceae;CHKCI002
Bacteria;Actinobacteria;Coriobacteriia;Coriobacteriales;Eggerthellaceae;Eggerthella
Bacteria;Actinobacteria;Coriobacteriia;Coriobacteriales;Eggerthellaceae;Slackia
Bacteria;Actinobacteria;Rubrobacteria;Rubrobacterales;Rubrobacteriaceae;Rubrobacter
Bacteria;Bacteroidetes;Bacteroidia;Bacteroidales;Rikenellaceae;Alistipes
Bacteria;Firmicutes;Bacilli;Lactobacillales;Carnobacteriaceae;Granulicatella
Bacteria;Firmicutes;Bacilli;Lactobacillales;Leuconostocaceae;Weissella
Bacteria;Firmicutes;Bacilli;Lactobacillales;Streptococcaceae;Streptococcus
Bacteria;Firmicutes;Clostridia;Clostridiales;Clostridiaceae 1;Sarcina
Bacteria;Firmicutes;Clostridia;Clostridiales;Eubacteriaceae;Eubacterium
Bacteria;Firmicutes;Clostridia;Clostridiales;Family XIII;Mogibacterium
Bacteria;Firmicutes;Clostridia;Clostridiales;Family XIII;[Eubacterium] nodatum group
Bacteria;Firmicutes;Clostridia;Clostridiales;Lachnospiraceae;Anaerostipes
Bacteria;Firmicutes;Clostridia;Clostridiales;Lachnospiraceae;Cellulosilyticum
Bacteria;Firmicutes;Clostridia;Clostridiales;Lachnospiraceae;Coprococcus 1
Bacteria;Firmicutes;Clostridia;Clostridiales;Lachnospiraceae;Epulopiscium
Bacteria;Firmicutes;Clostridia;Clostridiales;Lachnospiraceae;Fusicatenibacter
Bacteria;Firmicutes;Clostridia;Clostridiales;Lachnospiraceae;Lachnoclostridium
Bacteria;Firmicutes;Clostridia;Clostridiales;Lachnospiraceae;Lachnospira
Bacteria;Firmicutes;Clostridia;Clostridiales;Lachnospiraceae;Lachnospiraceae UCG-001
Bacteria;Firmicutes;Clostridia;Clostridiales;Lachnospiraceae;Lachnospiraceae UCG-003
Bacteria;Firmicutes;Clostridia;Clostridiales;Lachnospiraceae;Marvinbryantia
Bacteria;Firmicutes;Clostridia;Clostridiales;Lachnospiraceae;Sellimonas
Bacteria;Firmicutes;Clostridia;Clostridiales;Lachnospiraceae;Tyzzerella 3
Bacteria;Firmicutes;Clostridia;Clostridiales;Lachnospiraceae;[Eubacterium] ruminantium group
Bacteria;Firmicutes;Clostridia;Clostridiales;Lachnospiraceae;[Ruminococcus] gnavus group
Bacteria;Firmicutes;Clostridia;Clostridiales;Lachnospiraceae;[Ruminococcus] torques group
Bacteria;Firmicutes;Clostridia;Clostridiales;Lachnospiraceae;__
Bacteria;Firmicutes;Clostridia;Clostridiales;Peptostreptococcaceae;Intestinibacter
Bacteria;Firmicutes;Clostridia;Clostridiales;Peptostreptococcaceae;Paeniclostridium
Bacteria;Firmicutes;Clostridia;Clostridiales;Peptostreptococcaceae;Peptoclostridium
Bacteria;Firmicutes;Clostridia;Clostridiales;Ruminococcaceae;Faecalibacterium
Bacteria;Firmicutes;Clostridia;Clostridiales;Ruminococcaceae;Ruminococcaceae NK4A214 group

ESM Table 4. Region-specific gut microbiota predicted dietary habits*.

Food groups	Pearson r	*P*
Wheat	0.57 (0.42)	2.10E-242 (5.5E-121)
Rice	0.33 (0.24)	7.70E-71 (1.6E-37)
Pork	0.33 (0.27)	3.10E-70 (4.4 E-49)
Fish	0.21 (0.06)	2.80E-28 (1.4E-03)
Animal oil	0.2 (0.09)	5.80E-27 (1.9E-06)
Vegetable	0.2 (0.09)	4.60E-26 (4.9E-06)
Egg	0.19 (0.13)	4.10E-23 (2.4E-12)
Milk	0.15 (0.08)	9.40E-16 (2.6 E-05)
Poultry	0.13 (0.009)	7.10E-12 (1.4 E-06)
Fruit	0.1 (0.0037)	5.40E-08 (0.84)
Vegetable oil	0.1 (-0.007)	2.90E-07 (0.69)
Nuts	0.05 (-0.006)	7.00E-03 (0.74)

* We used the regression method from the LightGBM package to predict the levels of intake with the regionally differentiated genera. A 10-fold cross-validation (CV) predictive implementation was used to generate a genus-predicted intake values for each participant. Pearson correlation was calculated using actual dietary intake and predicted intake for the entire participants. The result of multiple imputation-based were presented in the brackets.

ESM Table 5. Prevalence of the glycemic-related gut microbiota.

Genera	Prevalence
Catenibacterium	63.80%
Erysipelatoclostridium	90.60%
Holdemanella	96.80%
Dialister	59.70%
Fusobacterium	22.00%
Sphingomonadaceae spp	12.00%
Delftia	22.50%
Halomonas	11.10%
Psychrobacter	24.40%
Mollicutes RF39 spp	19.20%
Paraprevotella	13.00%
Parabacteroides	53.60%
Enterococcus	96.20%
Family XIII AD3011 group	90.10%
[Eubacterium] brachy group	44.70%
Dorea	98.10%
Lachnospira	81.70%
Lachnospiraceae UCG-004	21.50%
Marvinbryantia	94.30%
[Ruminococcus] torques group	98.50%
Terrisporobacter	99.40%
Peptostreptococcaceae spp	18.10%
Butyricicoccus	88.90%
CAG-352	35.50%
Oscillibacter	24.50%

ESM Table 6. Prospective association of gut microbiota with glycemic traits*.

		Beta (95% CI)		
Gut microbiota	Glycemic trait	North	South	Overall
Atopobium	Fasting glucose	-0.064 (-0.14, 0.0075)	-0.034 (-0.088, 0.019)	-0.045 (-0.088, -0.0019)
Anaerofustis	HbA1c	-0.052 (-0.11, 0.011)	-0.032 (-0.079, 0.017)	-0.039 (-0.077, -0.001)
Defluviitaleaceae UCG-011	Fasting insulin	-0.0340 (-0.12, 0.05)	-0.056 (-0.11, 0.0019)	-0.049 (-0.097, -0.0012)

*We used linear mixed-effects model to examine the prospective association of gut microbiota with glycemic traits (fasting glucose, fasting insulin, HbA1C, HOMA-beta, and HOMA-IR), adjusting for the baseline glycemic traits, demographic, dietary and lifestyle confounders. Associations were expressed as the difference in glycemic traits (in SD units) per SD difference in each genus. The demographic, anthropometric, and lifestyle factors included age, sex, household income, marital status, self-reported educational level, place to live (rural or urban), urbanisation index, total energy intake, alcohol drinking, smoking, and physical activity.

ESM Fig. 1. Overview of the participants included in this analysis.

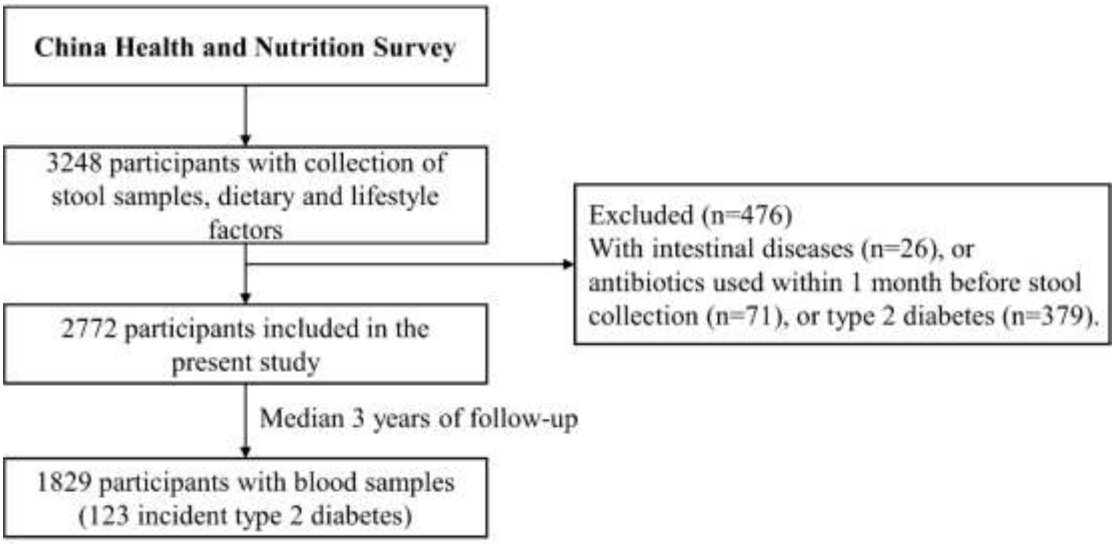

ESM Fig. 2. Prospective association of gut microbiota with the fasting glucose (A), HbA1c (B), fasting insulin (C) and HOMA IR (D). Linear mixed-effects model to examine the prospective association of gut microbiota with glycemic traits (fasting glucose, fasting insulin, HbA1C, and HOMA-IR), adjusting for the baseline glycemic traits, demographic, dietary and lifestyle confounders. Associations were expressed as the difference in glycemic traits (in SD units) per SD difference in each genus. The adjusted dietary factors including rice, wheat, fruit, vegetable, nuts, pork, poultry, milk, egg, fish, animal oil, and vegetable oil.

a

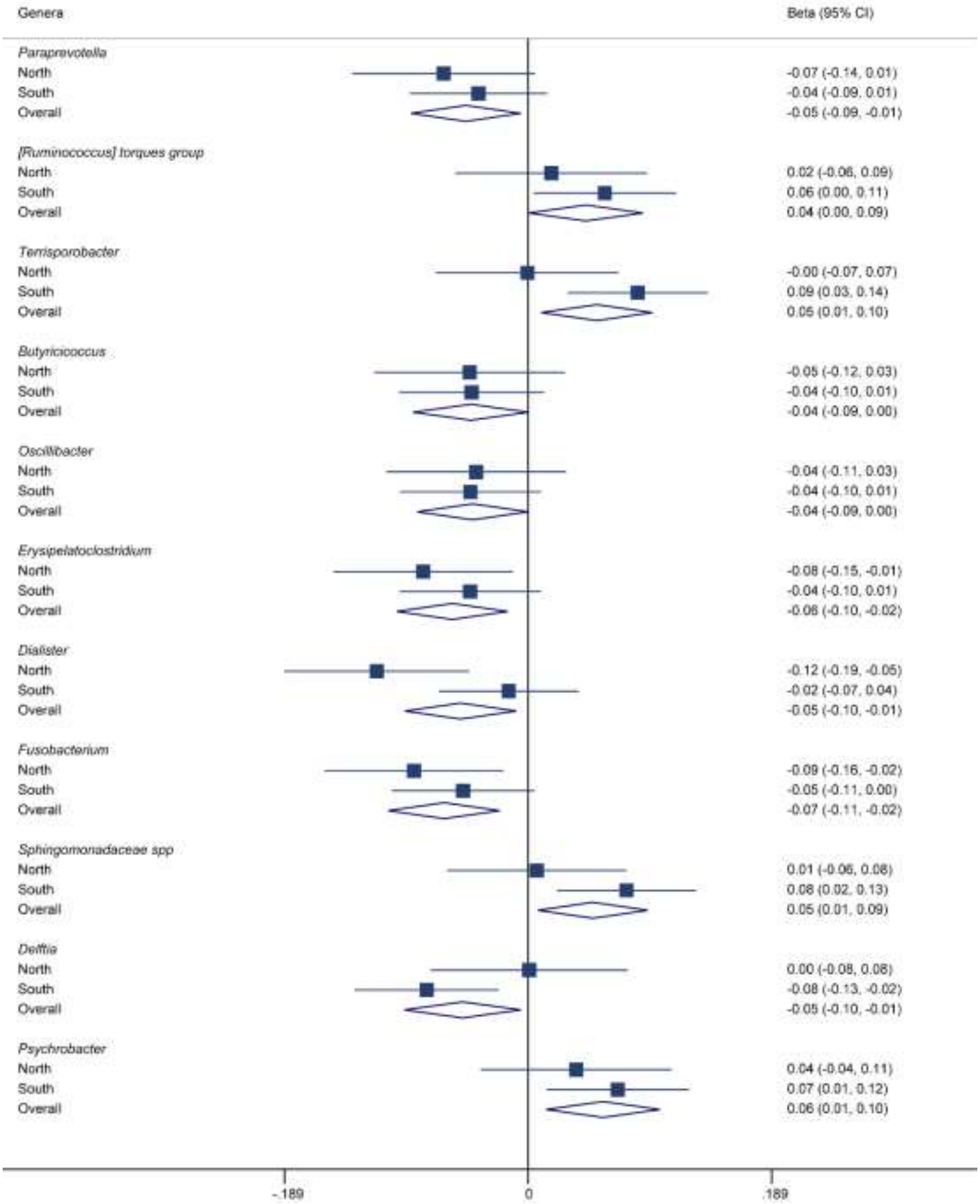
Genera
Beta (95% CI)
Paraprevotella
North -0.07 (-0.14, 0.01)
South -0.04 (-0.09, 0.01)
Overall -0.05 (-0.09, -0.01)
[Ruminococcus] torques group
North 0.02 (-0.06, 0.09)
South 0.06 (0.00, 0.11)
Overall 0.04 (0.00, 0.09)
Terrisporobacter
North -0.00 (-0.07, 0.07)
South 0.09 (0.03, 0.14)
Overall 0.05 (0.01, 0.10)
Butyricicoccus
North -0.05 (-0.12, 0.03)
South -0.04 (-0.10, 0.01)
Overall -0.04 (-0.09, 0.00)
Oscillibacter
North -0.04 (-0.11, 0.03)
South -0.04 (-0.10, 0.01)
Overall -0.04 (-0.09, 0.00)
Erysipelatoclostridium
North -0.08 (-0.15, -0.01)
South -0.04 (-0.10, 0.01)
Overall -0.06 (-0.10, -0.02)
Dialister
North -0.12 (-0.19, -0.05)
South -0.02 (-0.07, 0.04)
Overall -0.05 (-0.10, -0.01)
Fusobacterium
North -0.09 (-0.16, -0.02)
South -0.05 (-0.11, 0.00)
Overall -0.07 (-0.11, -0.02)
Sphingomonadaceae spp
North 0.01 (-0.06, 0.08)
South 0.08 (0.02, 0.13)
Overall 0.05 (0.01, 0.09)
Delftia
North 0.00 (-0.08, 0.08)
South -0.08 (-0.13, -0.02)
Overall -0.05 (-0.10, -0.01)
Psychrobacter
North 0.04 (-0.04, 0.11)
South 0.07 (0.01, 0.12)
Overall 0.06 (0.01, 0.10)
-.189
0
.189

b

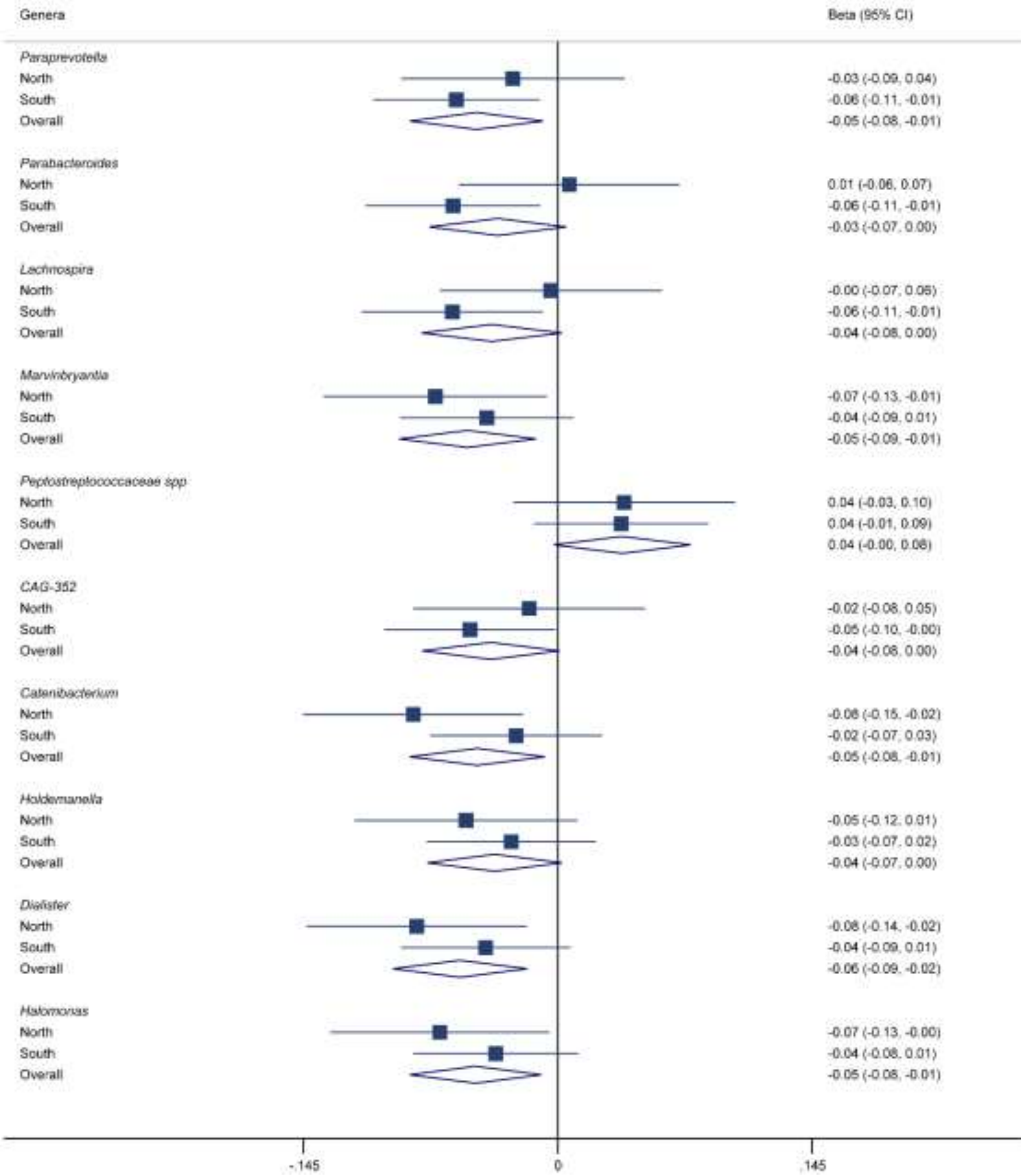
Genera
Beta (95% CI)
Paraprevotella
North
-0.03 (-0.09, 0.04)
South
-0.06 (-0.11, -0.01)
Overall
-0.05 (-0.08, -0.01)
Parabacteroides
North
0.01 (-0.06, 0.07)
South
-0.06 (-0.11, -0.01)
Overall
-0.03 (-0.07, 0.00)
Lachnospira
North
-0.00 (-0.07, 0.06)
South
-0.06 (-0.11, -0.01)
Overall
-0.04 (-0.08, 0.00)
Marvinbryantia
North
-0.07 (-0.13, -0.01)
South
-0.04 (-0.09, 0.01)
Overall
-0.05 (-0.09, -0.01)
Peptostreptococcaceae spp
North
0.04 (-0.03, 0.10)
South
0.04 (-0.01, 0.09)
Overall
0.04 (-0.00, 0.08)
CAG-352
North
-0.02 (-0.08, 0.05)
South
-0.05 (-0.10, -0.00)
Overall
-0.04 (-0.08, 0.00)
Catenibacterium
North
-0.08 (-0.15, -0.02)
South
-0.02 (-0.07, 0.03)
Overall
-0.05 (-0.08, -0.01)
Holdemanella
North
-0.05 (-0.12, 0.01)
South
-0.03 (-0.07, 0.02)
Overall
-0.04 (-0.07, 0.00)
Dialister
North
-0.08 (-0.14, -0.02)
South
-0.04 (-0.09, 0.01)
Overall
-0.06 (-0.09, -0.02)
Halomonas
North
-0.07 (-0.13, -0.00)
South
-0.04 (-0.08, 0.01)
Overall
-0.05 (-0.08, -0.01)
-.145
0
.145

c

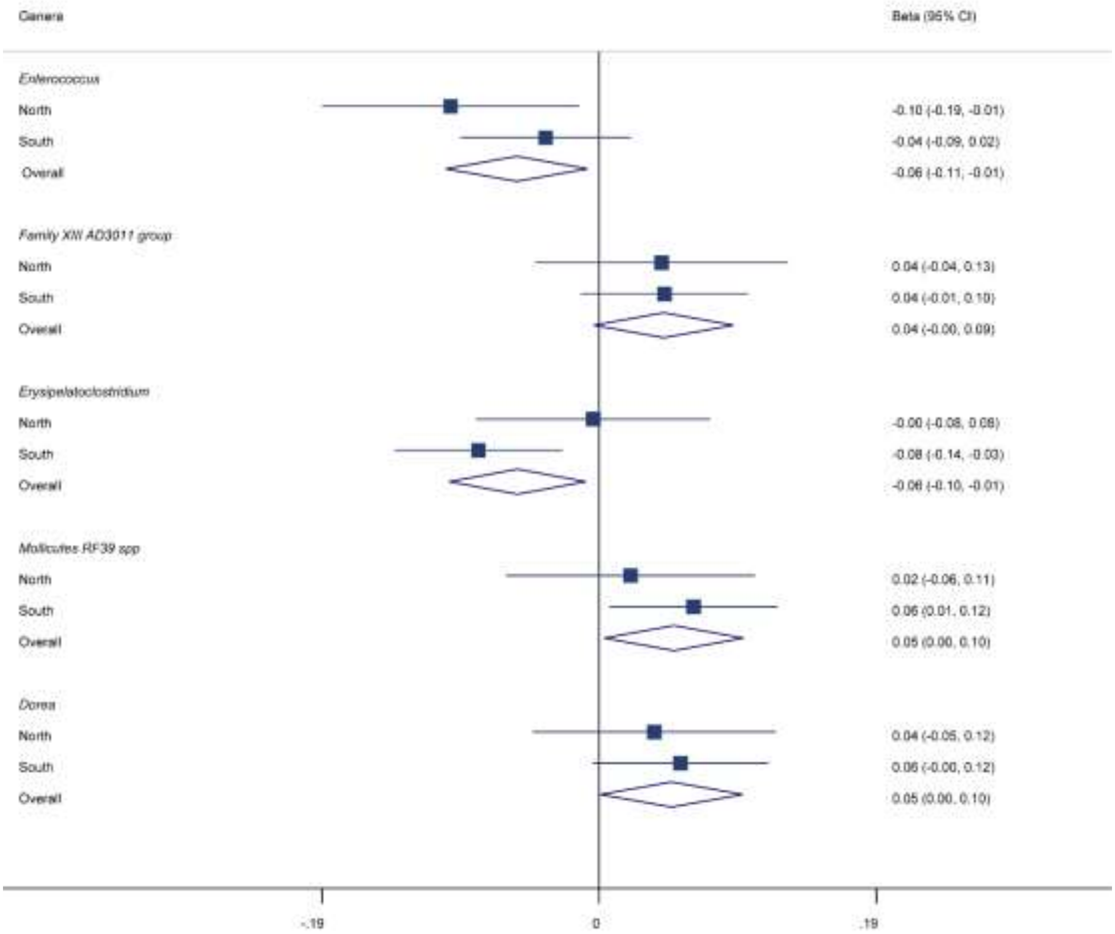

Genera
Beta (95% CI)
Enterococcus
North
-0.10 (-0.19, -0.01)
South
-0.04 (-0.09, 0.02)
Overall
-0.06 (-0.11, -0.01)
Family XIII AD3011 group
North
0.04 (-0.04, 0.13)
South
0.04 (-0.01, 0.10)
Overall
0.04 (-0.00, 0.09)
Erysipelatoclostridium
North
-0.00 (-0.08, 0.08)
South
-0.08 (-0.14, -0.03)
Overall
-0.06 (-0.10, -0.01)
Mollicutes RF39 spp
North
0.02 (-0.06, 0.11)
South
0.06 (0.01, 0.12)
Overall
0.05 (0.00, 0.10)
Dorea
North
0.04 (-0.05, 0.12)
South
0.06 (-0.00, 0.12)
Overall
0.05 (0.00, 0.10)
-.19
0
.19

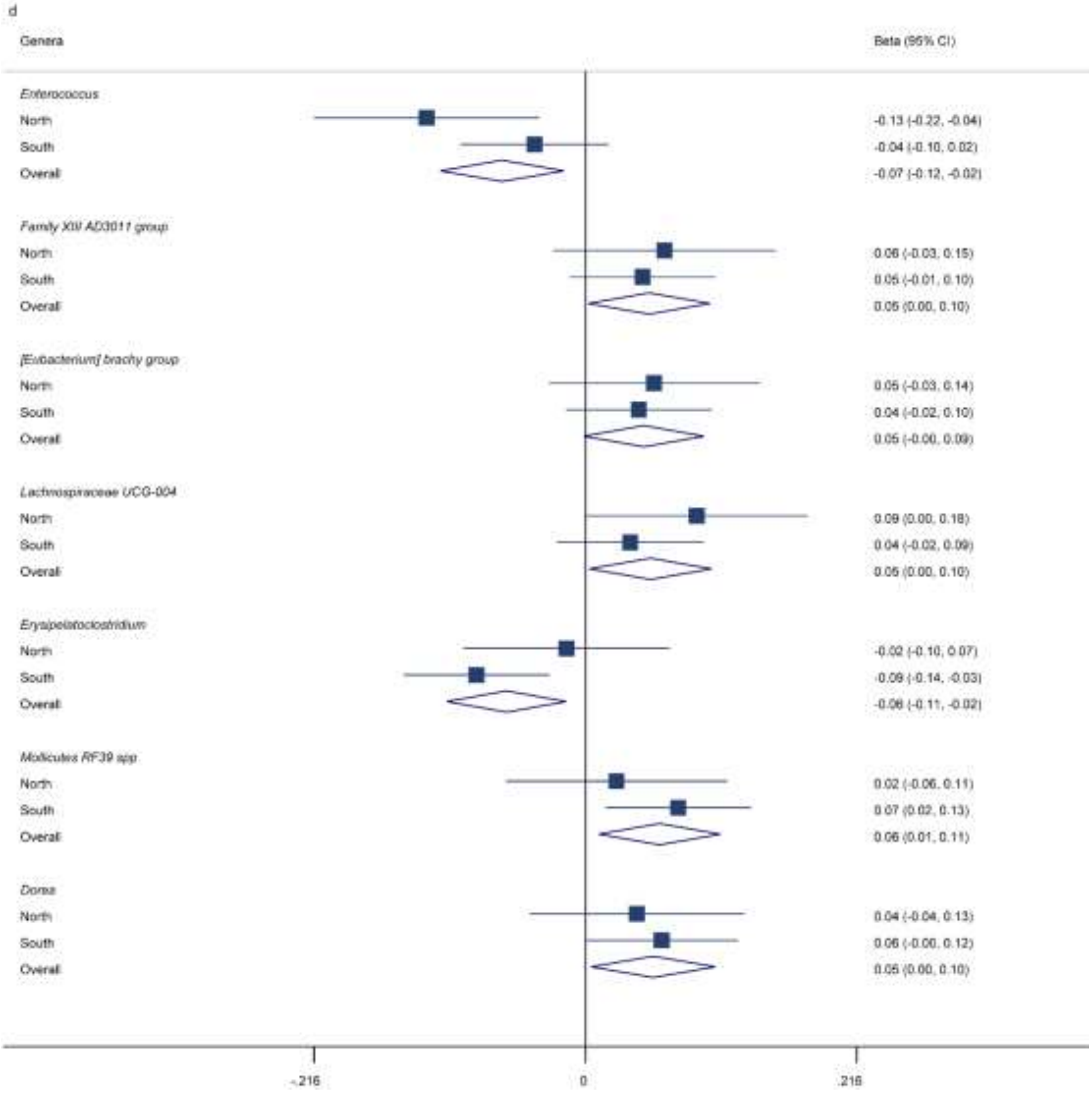
d
Genera
Beta (95% CI)
Enterococcus
North
-0.13 (-0.22, -0.04)
South
-0.04 (-0.10, 0.02)
Overall
-0.07 (-0.12, -0.02)
Family XIII AD3011 group
North
0.06 (-0.03, 0.15)
South
0.05 (-0.01, 0.10)
Overall
0.05 (0.00, 0.10)
[Eubacterium] brachy group
North
0.05 (-0.03, 0.14)
South
0.04 (-0.02, 0.10)
Overall
0.05 (-0.00, 0.09)
Lachnospiraceae UCG-004
North
0.09 (0.00, 0.18)
South
0.04 (-0.02, 0.09)
Overall
0.05 (0.00, 0.10)
Erysipelatoclostridium
North
-0.02 (-0.10, 0.07)
South
-0.09 (-0.14, -0.03)
Overall
-0.06 (-0.11, -0.02)
Mollicutes RF39 spp
North
0.02 (-0.06, 0.11)
South
0.07 (0.02, 0.13)
Overall
0.06 (0.01, 0.11)
Dorea
North
0.04 (-0.04, 0.13)
South
0.06 (-0.00, 0.12)
Overall
0.05 (0.00, 0.10)
-.216
0
.216

ESM Fig. 3. Association of dietary factors with glycemic traits. Linear regression was used to estimate the difference in glycemic traits (in standard deviation units) per-standard deviation change for continuous dietary with adjustment for the confounders and mutually adjusted for the other tested dietary or lifestyle factors. Model 1: The adjusted covariates including age, sex, BMI, total energy intake, household income, marital status, self-reported educational level, place to live (rural or urban), and urbanisation index. Model 2: Model 1 + gut bacterial composition (i.e., HMI).

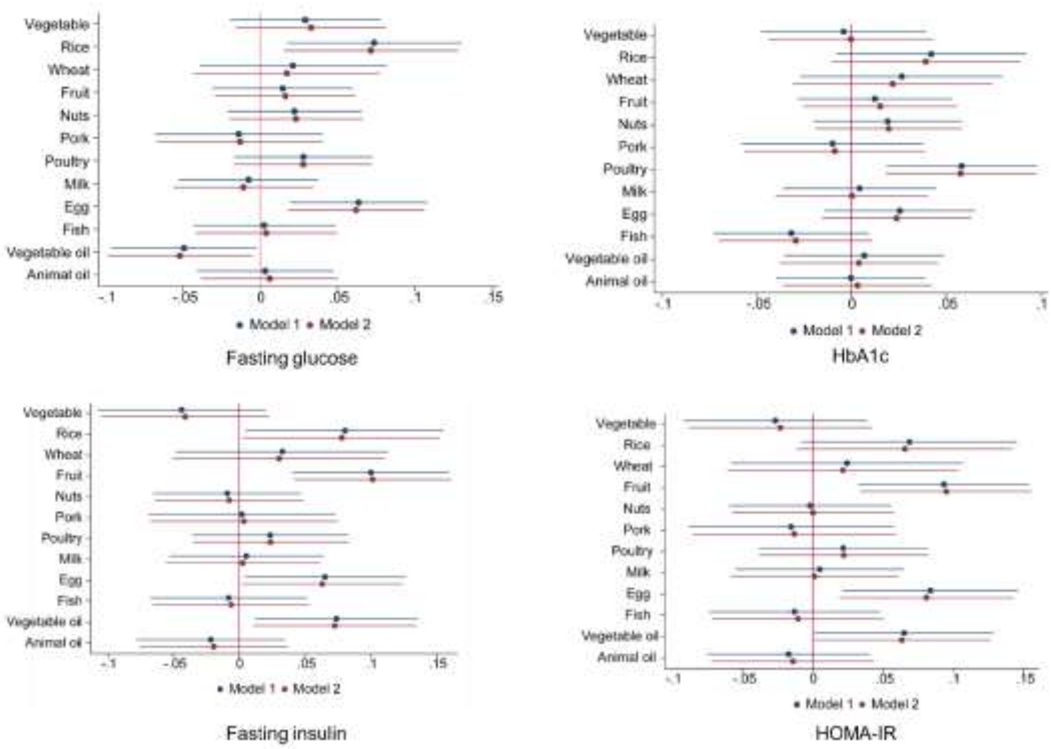

Supplement: Supplementary file 1 — (PDF 280 kb) [file 125_2022_5687_MOESM1_ESM.pdf]
